# Convalescent Plasma Therapy for COVID-19: A Graphical Mosaic of the Worldwide Evidence

**DOI:** 10.3389/fmed.2021.684151

**Published:** 2021-06-07

**Authors:** Stephen A. Klassen, Jonathon W. Senefeld, Katherine A. Senese, Patrick W. Johnson, Chad C. Wiggins, Sarah E. Baker, Noud van Helmond, Katelyn A. Bruno, Liise-anne Pirofski, Shmuel Shoham, Brenda J. Grossman, Jeffrey P. Henderson, R. Scott Wright, DeLisa Fairweather, Nigel S. Paneth, Rickey E. Carter, Arturo Casadevall, Michael J. Joyner

**Affiliations:** ^1^Department of Anesthesiology and Perioperative Medicine, Mayo Clinic, Rochester, MN, United States; ^2^Department of Health Sciences Research, Mayo Clinic, Jacksonville, FL, United States; ^3^Department of Anesthesiology, Cooper Medical School of Rowan University, Cooper University Health Care, Camden, NJ, United States; ^4^Department of Cardiovascular Medicine, Mayo Clinic, Jacksonville, FL, United States; ^5^Division of Infectious Diseases, Department of Medicine, Montefiore Medical Center, Albert Einstein College of Medicine, New York, NY, United States; ^6^Division of Infectious Diseases, Johns Hopkins University School of Medicine, Baltimore, MD, United States; ^7^Department of Pathology and Immunology, Washington University School of Medicine in St. Louis, St. Louis, MO, United States; ^8^Division of Infectious Diseases, Department of Medicine, Department of Molecular Microbiology, Washington University School of Medicine in St. Louis, St. Louis, MO, United States; ^9^Department of Cardiovascular Medicine, Human Research Protection Program, Mayo Clinic, Rochester, MN, United States; ^10^Department of Epidemiology and Biostatistics, Department of Pediatrics and Human Development, College of Human Medicine, Michigan State University, East Lansing, MI, United States; ^11^Division of Infectious Diseases, Johns Hopkins University School of Medicine, Baltimore, MD, United States

**Keywords:** convalescent plasma therapy, COVID-19, SARS-CoV-2, passive antibody transfer, Kaplan–Meier analysis

## Abstract

Convalescent plasma has been used worldwide to treat patients hospitalized with coronavirus disease 2019 (COVID-19) and prevent disease progression. Despite global usage, uncertainty remains regarding plasma efficacy, as randomized controlled trials (RCTs) have provided divergent evidence regarding the survival benefit of convalescent plasma. Here, we argue that during a global health emergency, the mosaic of evidence originating from multiple levels of the epistemic hierarchy should inform contemporary policy and healthcare decisions. Indeed, worldwide matched-control studies have generally found convalescent plasma to improve COVID-19 patient survival, and RCTs have demonstrated a survival benefit when transfused early in the disease course but limited or no benefit later in the disease course when patients required greater supportive therapies. RCTs have also revealed that convalescent plasma transfusion contributes to improved symptomatology and viral clearance. To further investigate the effect of convalescent plasma on patient mortality, we performed a meta-analytical approach to pool daily survival data from all controlled studies that reported Kaplan–Meier survival plots. Qualitative inspection of all available Kaplan–Meier survival data and an aggregate Kaplan–Meier survival plot revealed a directionally consistent pattern among studies arising from multiple levels of the epistemic hierarchy, whereby convalescent plasma transfusion was generally associated with greater patient survival. Given that convalescent plasma has a similar safety profile as standard plasma, convalescent plasma should be implemented within weeks of the onset of future infectious disease outbreaks.

## Introduction

In response to the coronavirus disease 2019 (COVID-19) pandemic caused by the novel human severe acute respiratory syndrome coronavirus 2 (SARS-CoV-2), convalescent plasma has been used worldwide to treat hospitalized patients and prevent progression of disease in non-hospitalized patients (1–4). Due to the limited number of therapeutic alternatives for COVID-19, widespread optimism for antibody therapy has also led to the commercial production of other immunoglobulin therapies, including monoclonal antibodies and hyperimmune products (5, 6). However, despite global usage, clinicians and researchers have struggled to reach consensus regarding the clinical utility of convalescent plasma. Although signatures of efficacy have emerged consistently from worldwide matched-control studies (7), there remains a paucity of data from large randomized clinical trials (RCTs) demonstrating efficacy. Recent reviews and meta-analyses have also generated disharmonious conclusions regarding the effect of convalescent plasma on COVID-19 patient outcomes, such as mortality and clinical improvement. A pragmatic meta-analysis by our group that included data from both RCTs and matched-control studies demonstrated a mortality reduction attributed to convalescent plasma in COVID-19 patients (8). Conversely, recent reviews that adhered to stricter study inclusion rules and aggregated only RCT data concluded that either convalescent plasma had no effect on mortality or that insufficient evidence exists to determine efficacy (9, 10). In addition, data emerging from the RECOVERY Trial in the United Kingdom showed no survival benefit of convalescent plasma in its overall cohort that was severely ill but demonstrated trends toward efficacy when plasma was transfused to patients who were earlier in the disease course and did not require supplemental oxygen or corticosteroids (11). These contradictory findings present a challenge for physicians in deciding the therapeutic strategy for COVID-19 patients.

We argue that during a global health emergency like a pandemic, evidence originating from multiple levels of the epistemic evidence hierarchy should be used to inform contemporary policy and healthcare decisions. Such an approach is needed for a complex therapy like convalescent plasma where efficacy depends on the quality of the plasma, the timing of administration, and the immunological status of the patient (12). Thus, this graphical review aims to showcase the mosaic of worldwide evidence related to convalescent plasma therapy for COVID-19.

## Principles of Convalescent Plasma Therapy

Briefly, convalescent plasma represents a form of passive antibody therapy that relies on the transfer of pathogen-specific antibodies from a recovered patient for the purpose of preventing or treating disease (13). Unlike vaccines and monoclonal antibodies, convalescent plasma therapy requires limited development or infrastructure, requiring no more than the availability of disease survivors willing to donate plasma and standard blood collection infrastructure to collect and distribute convalescent plasma (14). With no vaccines or monoclonal antibodies available at the onset of the COVID-19 pandemic, convalescent plasma was an immediately deployable option (14, 15). In addition, for this reason, convalescent plasma can be readily used in low resource settings across the globe.

Convalescent plasma is also adaptable to changing conditions. As variant SARS-CoV-2 strains continue to emerge, convalescent plasma donated by survivors of infections with variant strains represents an immediately deployable therapeutic for patients identified with a variant infection, whereas other immune therapies may require (re)development to more specifically target new viral strains (16–19).

To effectively neutralize SARS-CoV-2 and confer clinical benefit, convalescent plasma must adhere to the three fundamental principles of passive antibody therapy (12). Convalescent plasma *must* (20–22):

i) Contain specific antibodies against the pathogen, the SARS-CoV-2 virus
ii) Contain a sufficient level of anti-SARS-CoV-2 antibody, and
iii) Be transfused prophylactically or early in the disease course.

## Convalescent Plasma for Previous Respiratory Viral Outbreaks

The widespread use of convalescent plasma in the COVID-19 pandemic was founded on its rich history of efficacy against human respiratory viral infections. Indeed, the first Nobel Prize in Physiology or Medicine was awarded for the discovery of convalescent plasma (serum) therapy for diphtheria (15). Since the late nineteenth century, convalescent plasma has been used to mitigate several outbreaks caused by human respiratory viruses. A meta-analysis of eight studies (*n* = 1,703 patients) found that convalescent plasma reduced the absolute risk of death by 21% in patients with H1N1 viral infections during the 1918 influenza pandemic (23). Subgroup analysis of patients transfused with convalescent plasma within 3 days of illness onset demonstrated a 41% lower risk of death compared to patients transfused four or more days after illness onset, highlighting an important role for timely convalescent plasma transfusion (23). Convalescent plasma has also been associated with positive clinical outcomes in recent outbreaks caused by other coronaviruses, including the 2001 SARS1 epidemic and the 2012 Middle East Respiratory Syndrome (MERS) (24–26). For instance, in 80 patients diagnosed with SARS1, patients transfused with convalescent plasma within 2 weeks of illness onset were more likely to be discharged by day 22 of hospitalization than patients treated later in the disease course (24). Although most of the historical evidence for convalescent plasma emerged from retrospective matched-control designs and single-arm studies, the favorable efficacy data and positive safety signals provided strong precedent for rapid implementation at the onset of the COVID-19 pandemic (14, 20).

## Convalescent Plasma Therapy for COVID-19: Experimental Evidence

In the context of the COVID-19 pandemic, the anticipated primary mechanism for the clinical benefit of convalescent plasma immunotherapy is SARS-CoV-2 viral neutralization (27, 28). Virus neutralization occurs when antibodies bind to the spike protein and prevent binding to the host cellular receptors. In addition to viral neutralization, convalescent plasma includes antibodies that mediate three other antiviral functions against SARS-CoV-2: (i) complement activation, (ii) antibody-dependent cellular cytotoxicity, and (iii) phagocytosis (29). The antiviral effect of convalescent plasma is supported by RCTs and observational studies, which have consistently shown a reduction in viral load following transfusion (3, 27, 30).

COVID-19 pathogenesis begins with an early viral phase that can progress to a life-threatening inflammatory phase (31). The viral phase is characterized by SARS-CoV-2 virus replication that is accompanied by variable symptoms and triggers an endogenous antibody response around days 10–12 of infection (32). Some individuals may progress to an inflammatory phase that may clear the virus but impairs pulmonary gas exchange and in some cases causes respiratory failure and death (31, 33, 34). Thus, early convalescent plasma transfusion during the viral phase is effective because viral neutralization prevents disease progression to the severe inflammatory phase. Consistent with this view, convalescent plasma administration in COVID-19 is followed by reduction in markers of inflammation (35, 36). A synthesis of these observations suggests that early administration of convalescent plasma reduces viral burden through antibody-mediated antiviral effects, which in turn prevents inflammation promoting a survival benefit by improving oxygen exchange in the lung.

### Animal Studies

During the COVID-19 pandemic, several animal studies performed in parallel with human clinical investigations have demonstrated convalescent plasma efficacy in experimental SARS-CoV-2 infection. For example, convalescent serum from Syrian hamsters elicited an antiviral effect when administered to infected animals (37). Similarly, among SARS-CoV-2-infected green monkeys, transfusion of convalescent plasma from the same species reduced lung pathology, viral shedding, and inflammation (38). In addition, administration of human convalescent plasma protected mice expressing the human ACE2 receptor from SARS-CoV-2 infection (39). These studies illustrate that animal and human convalescent plasma contains virus-neutralizing antibodies that mediate protective effects in animal models of SARS-CoV-2 infection.

## Convalescent Plasma Therapy for COVID-19: Mosaic of Clinical Evidence

To date, 11 RCTs (11, 27, 30, 40–47) and 24 matched-control studies (3, 4, 48–69) have investigated convalescent plasma therapy for COVID-19. Of these studies, 16 presented survival data using a Kaplan–Meier diagram (11, 27, 41, 43, 46, 47, 50, 52, 59–62, 64, 69–71). To investigate the impact of convalescent plasma on COVID-19 patient survival over time, we extracted daily survival data from all available Kaplan–Meier diagrams using an online data extraction tool (WebPlotDigitizer v4.4, Pacifica, CA, USA). Kaplan–Meier survival plots were replotted for each study. Aggregate Kaplan–Meier survival plots were computed by pooling the derived number of deaths per day from the daily survival rate and reported number of patients at risk. For aggregate survival plots, 95% confidence intervals were calculated by Greenwood's formula (72). This meta-analytical approach provides greater temporal resolution of survival data compared to conventional meta-analyses that rely on the overall survival rate at the end of the study period (73). Figures were generated with R software (R Core Team, Vienna, Austria) and GraphPad Prism (GraphPad Software, San Diego, CA, USA).

**Figure 1:**
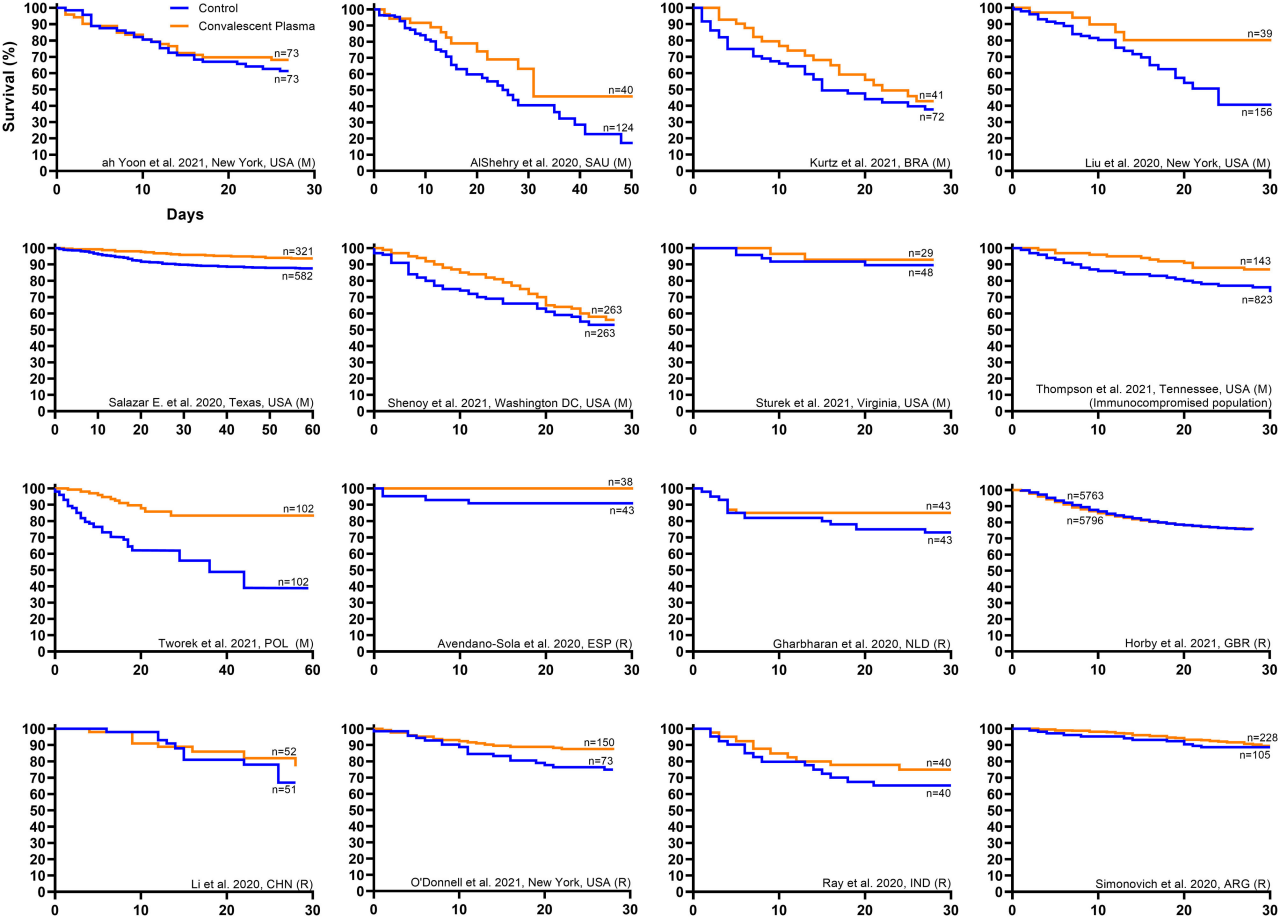
Kaplan–Meier survival plots from worldwide controlled studies investigating convalescent plasma therapy for coronavirus disease 2019 (COVID-19). Available Kaplan–Meier survival data from worldwide controlled studies [*n* = 7 randomized clinical trials (11, 27, 41, 43, 46, 47, 71) and *n* = 9 matched-control studies (50, 52, 59–62, 64, 69, 70)] was extracted and replotted. Each panel represents data from one study, where the orange line represents the survival of the cohort transfused with convalescent plasma, and the blue line represents the survival of the cohort that received standard of care or placebo transfusion. The label for each study provides the first author name, location, and study type, where matched-control studies are denoted by (M) and randomized clinical trials are denoted by (R). For each study, sample sizes from the number at risk at study onset are presented for the treatment and control cohorts. No statistical analyses were performed. *Interpretation:* Qualitative inspection of the Kaplan–Meier survival data from 16 controlled studies revealed a directionally consistent pattern whereby convalescent plasma transfusion was associated with greater patient survival compared to non-transfused patients.

**Figure 2:**
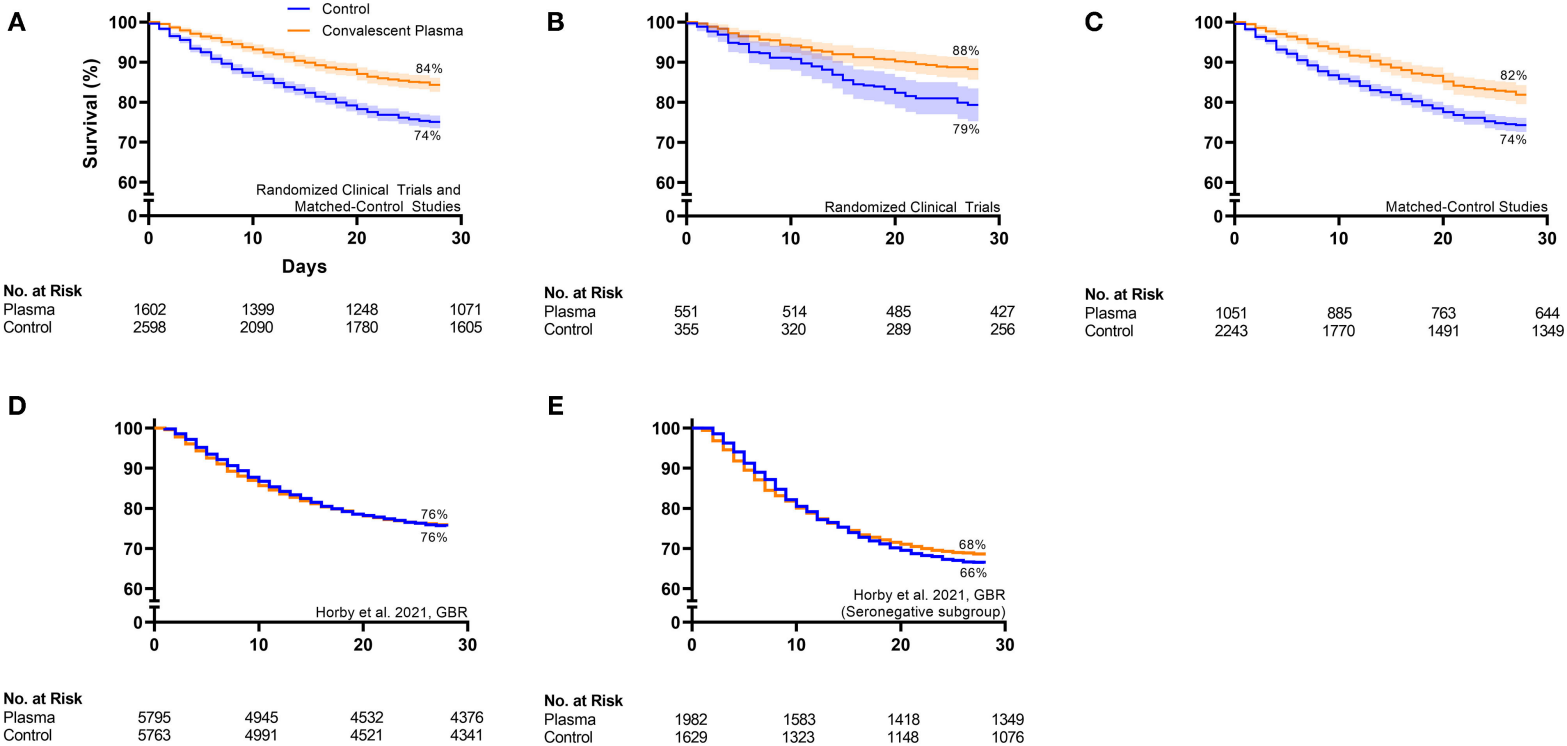
Aggregate Kaplan–Meier survival plots from all available controlled studies investigating convalescent plasma therapy for COVID-19. **(A)** Pools all controlled studies [*n* = 6 randomized clinical trials (11, 27, 41, 43, 47, 71, 74) and *n* = 9 matched-control studies (50, 52, 59–62, 64, 69, 70)] with available Kaplan–Meier survival data except for the large randomized clinical trial, the RECOVERY Trial. **(B)** Pools randomized clinical trials (*n* = 6) with available data except for the RECOVERY Trial. **(C)** Pools matched-control studies (*n* = 9) with available data. **(D)** Shows the RECOVERY Trial data from the overall cohort, and **(E)** shows the RECOVERY Trial data from the subgroup of patients that were seronegative at the time of plasma transfusion. Available 28-day Kaplan–Meier survival data from worldwide controlled studies was extracted, pooled, and replotted. For each panel, the orange line represents the survival of the cohort transfused with convalescent plasma, and the blue line represents the survival of the cohort that received standard of care or placebo transfusion. Error bands represent 95% confidence intervals. The 28-day survival (%) for the aggregate convalescent plasma cohort and the aggregate control cohort is presented. The table at the bottom of each panel presents the number at risk on the day of enrollment or randomization, day 10, day 20, and day 28 for the aggregate convalescent plasma cohort and the aggregate control cohort. *Interpretation:* The aggregate survival data from **(A)** all controlled studies except for the RECOVERY Trial, **(B)** all randomized clinical trials except for the RECOVERY Trial, and **(C)** all matched-control studies revealed a consistent pattern whereby convalescent plasma transfusion was associated with greater patient survival compared to non-transfused patients. **(D)** The overall RECOVERY Trial cohort demonstrated no survival benefit of convalescent plasma. **(E)** The modest efficacy signal from the RECOVERY Trial subgroup of seronegative patients supports the concept that early plasma transfusion reduces patient mortality.

Qualitative inspection of the available Kaplan–Meier survival data from 16 controlled studies revealed a directionally consistent pattern whereby convalescent plasma transfusion was associated with greater patient survival compared to non-transfused patients (Figure 1) (11, 27, 41, 43, 46, 47, 50, 52, 59–62, 64, 69–71). Pooling all available Kaplan–Meier survival data showed a 14% relative improvement in COVID-19 patient 28-day survival associated with convalescent plasma (84 vs. 74%) (Figure 2A). We offer the caveat that due to the large sample size of the RECOVERY Trial (11), these data were not included in this aggregation but reported separately (Figure 2D). The RECOVERY Trial found no difference in 28-day mortality between COVID-19 patients treated with convalescent plasma and non-transfused patients (24 vs. 24%) (11). The general survival benefit associated with convalescent plasma has been observed worldwide including studies that were heterogenous for health system type and infectious disease or critical care infrastructure, timing relative to pandemic onset, convalescent plasma antibody titer and volume, and patient disease severity (Supplementary Figure 1). This consistency of evidence across nations with different health systems supports the notion that convalescent plasma therapy is effective against COVID-19.

### Randomized Clinical Trials

Signatures of convalescent plasma efficacy have emerged from some but not all RCTs. Inspection of available Kaplan–Meier survival data from seven published RCTs revealed a directionally consistent pattern whereby convalescent plasma appeared to improve COVID-19 patient survival compared to patients that received standard of care (Figure 1) (11, 27, 41, 43, 46, 47, 71). Pooling these available survival data demonstrated a 11% relative improvement in 28-day survival associated with convalescent plasma compared to control (88 vs. 79%) (Figure 2B). While the effect of convalescent plasma on mortality was found to be statistically significant in only two RCTs (42, 71), it is important to highlight that signals of efficacy have emerged despite several limitations, including trials that (i) were underpowered due to declining local infection rates and early termination (27, 43), (ii) transfused convalescent plasma units with unknown or low anti-SARS-CoV-2 antibody levels (30), (iii) transfused severely ill patients that had progressed to severe COVID-19 (11, 27), and (iv) used prepositioned plasma units, which may not have accounted for SARS-CoV-2 variants (18, 19). Transfusion of low antibody titer plasma to patients with severe COVID-19 reduces the opportunity to achieve the survival benefit associated with convalescent plasma. Due to the large sample size of the RECOVERY Trial [*n* = 11,558; (11)], these data were not included in the aggregate RCT Kaplan–Meier plot (Figure 2B) but reported separately (Figure 2D). The RECOVERY Trial likely did not find an effect of convalescent plasma in their overall cohort due to the large proportion of severely ill patients (24% overall mortality) who were treated late in the disease course (median time to transfusion, 9 days). Corticosteroids, which interfere with antibody function, were used in more than 90% of study patients in both arms (11).

Nonetheless, some signals of efficacy emerged from subgroup analyses of the RECOVERY Trial data (11). First, in four likely overlapping groups of patients characterized as representing earlier disease, significant or near-significant lowering of mortality was seen with convalescent plasma: among patients transfused within 7 days of symptom onset [odds ratio, 0.92 (95% confidence interval, 0.83–1.03)], patients not receiving corticosteroids [odds ratio, 0.78 (95% confidence interval, 0.58–1.05)], patients not on supplemental oxygen [odds ratio, 0.83 (95% confidence interval, 0.58–1.18)], and patients without an endogenous anti-SARS-CoV-2 antibody response at time of transfusion [see Figure 2E—odds ratio, 0.94 (95% confidence interval: 0.84–1.06)] (11). These trends toward a survival benefit with early plasma transfusion are consistent with the RCT by Libster et al., which observed a ~50% reduction in progression to severe COVID-19 respiratory disease when mildly ill patients were transfused with convalescent plasma screened for appropriate level of antibody and a 73% reduction when plasma with antibody above the median was used (45).

Not all meta-analytical approaches support the impact of convalescent plasma on patient survival. A recent Cochrane review and meta-analysis that was restricted to only a few RCTs found insufficient evidence to draw conclusions regarding plasma efficacy (10). In addition, a meta-analysis of 10 RCTs by Janiaud et al. found that convalescent plasma was not associated with improved survival among COVID-19 patients (9). This latter meta-analysis was strongly affected by RECOVERY Trial data, which had a statistical weight of 90%. By contrast, our pragmatic meta-analysis of RCT data showed that overall in all studies, patients transfused with convalescent plasma exhibited reduced mortality rates compared to non-transfused COVID-19 patients (11 vs. 16% mortality; OR, 0.65; 95% CI, 0.43, 0.98, *P* = 0.04) (8). For the interpretation of these results, we offer the caveat that our prior analyses excluded the trial by Agarwal et al. (30) due to the low plasma quality and was published before the release of the RECOVERY Trial data (11). Completion of several ongoing RCTs designed in accordance with the key principles of passive antibody therapy will provide more comprehensive data regarding the effect of convalescent plasma on COVID-19 patient survival.

Even among trials that did not find a survival benefit, RCTs identified positive effects of convalescent plasma on other clinical outcomes including symptomatology, oxygen requirements, and viral clearance. For example, Agarwal et al. found that a significantly greater proportion of transfused patients exhibited improved viral clearance and resolution of shortness of breath and fatigue within 1 week of randomization (30). Similarly, among patients with severe disease, Li et al. observed that transfused patients exhibited greater viral clearance and shorter times to reductions in supplemental oxygen and hospital discharge (27). Other RCTs have made similar observations (40–43, 45, 46). These additional clinical outcomes compliment positive safety data to support convalescent plasma therapy for patients hospitalized with COVID-19 (75, 76).

### Matched-Control Studies

In contrast to RCTs, matched-control studies have generally observed a survival benefit associated with convalescent plasma therapy. Inspection of available Kaplan–Meier survival data from nine published matched-control studies showed a consistent pattern whereby convalescent plasma appeared to improve COVID-19 patient survival compared to non-transfused patients (Figure 1) (50, 52, 59–62, 64, 69, 70). Combining these available survival data demonstrated an 11% relative improvement in 28-day survival associated with convalescent plasma compared to control patients (82 vs. 74%) (Figure 2C). Our recent meta-analysis that aggregated data from 20 matched-control studies supports these findings (8). These analyses indicated that patients transfused with convalescent plasma exhibited a 43% relative reduction in mortality compared to patients receiving standard treatments (21 vs. 29% mortality; OR, 0.57; 95% CI, 0.45, 0.72; *P* < 0.001) (8). The contrasting mortality benefit between matched-control studies and larger multicenter RCTs raises the possibility that locally sourced plasma administered shortly after donation may increase convalescent plasma efficacy against local variants in real time as they emerge (17). Compared to RCTs, matched-control studies are inherently predisposed to greater bias risk (77). However, several studies observed convalescent plasma efficacy using rigorous propensity matching for key variables such as age, sex, disease severity and oxygen requirements, and comorbidities (52, 64, 69, 70). For example, a large propensity-matched study by Salazar and colleagues found that transfusion of high-titer convalescent plasma within 44 h of hospitalization reduced mortality among patients hospitalized with COVID-19 (64).

### Other Clinical Evidence

The evidence mosaic for convalescent plasma efficacy also includes data from dose–response studies and case series of vulnerable immunocompromised patient populations. On the basis that anti-SARS-CoV-2 neutralizing antibodies represent the primary mechanism for plasma efficacy, we previously examined the dose–response relationship between antibody titer level and 30-day mortality among 3,082 transfused patients via the United States Expanded Access Program for convalescent plasma (78). Transfusion of higher titer plasma was associated with lower 30-day mortality than transfusion of plasma with lower antibody titers (22 vs. 30%) (78). Convalescent plasma conferred a survival benefit only in patients that were transfused earlier in the disease course, as this association was not observed in patients that were mechanically ventilated at the time of transfusion (78). Similarly, in the Argentine Expanded Access Program experience (*n* = 4,719), patients transfused with convalescent plasma within 3 days of hospitalization demonstrated a 65% reduction in mortality compared to patients transfused after 7 days of hospitalization (2).

Evidence of efficacy has also emerged from studies of convalescent plasma therapy in immunocompromised patients who cannot generate an innate immune response (79). In a case series of three COVID-19 patients with X-linked agammaglobulinemia (XLA) who required supplemental oxygen, convalescent plasma transfusion was associated with rapid improvements in oxygen requirements and symptomatology in all patients (80). These data align with observations from the RECOVERY Trial subgroup analysis, which demonstrated a trend toward a reduction in mortality associated with convalescent plasma transfusion among patients that did not generate a SARS-CoV-2 antibody response (11). These observations also provide a unique “experiment of nature” for convalescent plasma, as the rapid recovery of these patients was not confounded by endogenous production of neutralizing antibodies.

## Clinical Limitations of Convalescent Plasma Therapy for COVID-19

Challenges associated with convalescent plasma therapy likely limit clinical evidence and interpretation of the mosaic of evidence. First, the therapeutic dose *per se* of convalescent plasma that imposes viral neutralization and improves the recipient's clinical condition is unknown and represents a highly complicated concept. In addition, a “gold standard” serological assay to detect antibodies to SARS-CoV-2 has not been identified, and the assays in use are investigational (81). Thus, some studies likely failed to identify a clinical benefit of convalescent plasma due to low antibody levels, and between-trial differences in both antibody levels and assays may contribute to variability in outcomes between trials. Given the limitations of low antibody levels, the US Food and Drug Administration (FDA) now requires use of convalescent plasma with higher antibody levels, thereby eliminating the use of low antibody convalescent plasma (1).

Second, the precise therapeutic window of convalescent plasma that corresponds to antiviral effects is unknown but convalescent plasma likely has limited effects late in the COVID-19 disease course, for example, among patients that require mechanical ventilation. Some studies that reported no effect of convalescent plasma transfused patients that were in advanced stages of COVID-19 respiratory failure attributed to an excessive inflammatory response (11, 27, 30). These results should not be surprising and may be interpreted as confirming the limitations of the therapeutic window of convalescent plasma rather than interpreted as “null findings” with respect to therapeutic effectiveness of plasma.

Evidence emerging from RCTs suggests that the therapeutic window of convalescent plasma coincides with that of the antiviral remdesivir, which was associated with greater patient recovery when administered prior to transition to high-flow oxygen and mechanical ventilation (82). Two other experimental COVID-19 therapies may be used to treat patients outside the therapeutic window of convalescent plasma. Prior to hospitalization, COVID-19 patients may receive monoclonal antibodies, which reduce both viral load and the time to symptom resolution in non-hospitalized individuals with COVID-19 (83, 84). Upon progression to severe COVID-19 requiring mechanical ventilation, dexamethasone represents a promising experimental therapy for stemming the inflammatory phase of COVID-19 and improving patient survival (85).

## Conclusions and Future Directions

Currently, a mosaic of evidence from varying epistemic levels exists for convalescent plasma therapy. While most RCTs indicate that convalescent plasma confers no survival benefit when used in severely ill hospitalized COVID-19 patients, these same trials have identified clinical improvements, such as viral clearance and reduced supplemental oxygen, following plasma transfusion. In addition, although two of the largest RCTs found no effect of convalescent plasma, these trials were limited by low plasma quality (30) or a high proportion of severely ill patients concomitantly treated with corticosteroids [RECOVERY Trial (11)], which can interfere with antibody function. In contrast, worldwide matched-control studies have consistently found that convalescent plasma improves COVID-19 patient survival. Qualitative inspection of individual and aggregate Kaplan–Meier survival curves from RCTs and matched-control studies reveals a directionally consistent pattern, suggesting convalescent plasma efficacy in COVID-19 patients. Importantly, worldwide studies have consistently found that convalescent plasma therapy is as safe as standard plasma. Thus, while individual lines of evidence forming the mosaic each have foibles, such as potential bias in the observational studies and late usage of low titer plasma in some RCTs, the composite mosaic encourages the use and continued study of convalescent plasma. In this regard, there have been five major infectious disease outbreaks in 21 years, namely, SARS, MERS, Zika virus, Ebola, and COVID-19, and convalescent plasma therapy was used for all but Zika virus. Convalescent plasma epitomizes a “common sense” therapeutic, which, if implemented rapidly in future infectious disease outbreaks and in a manner that abides by the core principles of passive antibody therapy, can continue to serve as a stopgap therapy until more effective strategies are identified.

## Author Contributions

SK, JS, AC, and MJ conceived and designed the study. SK, KS, JS, PJ, RC, AC, and MJ analyzed the data and generated figures. All authors reviewed, critically revised, and approved the final version of the manuscript.

## Conflict of Interest

The authors declare that the research was conducted in the absence of any commercial or financial relationships that could be construed as a potential conflict of interest.
