# Supplementary material for: Convalescent Plasma Therapy for COVID-19: A Graphical Mosaic of the Worldwide Evidence
Source: Front Med (Lausanne). 2021 Jun 7;8:684151. doi: 10.3389/fmed.2021.684151 (PMC8215127; doi:10.3389/fmed.2021.684151)

**Supplementary Appendix**

**Supplement to: Klassen SA, Senefeld JW, Senese KA, et al. Convalescent Plasma Therapy for COVID-19: A Graphical Mosaic of the Worldwide Evidence**

**Figure S1. Global distribution of studies investigating convalescent plasma therapy for COVID-19.** Chloropleth map displaying the number of worldwide convalescent plasma studies by country, with lower numbers of studies displayed in yellow and higher numbers of studies displayed in purple. This map includes randomized clinical trials, matched-control studies, and case-series or reports.

*Interpretation:* The general survival benefit and clinical improvement associated with convalescent plasma has been observed in over 30 countries worldwide including studies that were heterogenous for health system type and infectious disease or critical care infrastructure, timing relative to pandemic onset, convalescent plasma antibody titer and volume, and patient disease severity. Thus, we suspect this global consistency of evidence supports the notion that convalescent plasma therapy is effective against COVID-19


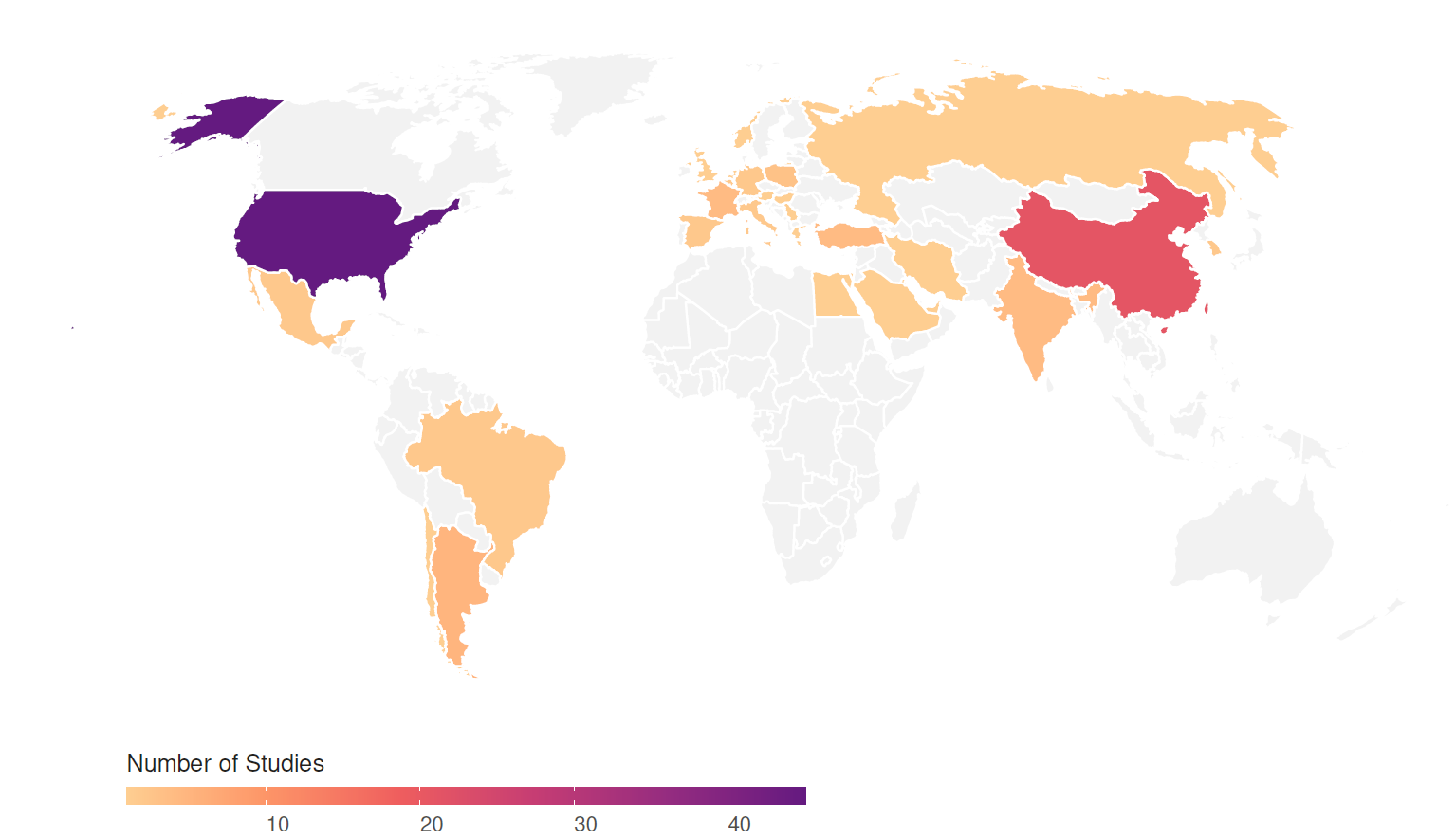

Supplement: Supplementary file 1 [file Data_Sheet_1.DOCX]
